# Mindfulness-Based Interventions During Pregnancy: a Systematic Review and Meta-analysis

**DOI:** 10.1007/s12671-017-0726-x

**Published:** 2017-04-17

**Authors:** Anjulie Dhillon, Elizabeth Sparkes, Rui V. Duarte

**Affiliations:** 1 School of Psychology, Sport and Exercise, Staffordshire University, Stoke-on-Trent, UK; 2 Faculty of Health and Life Sciences, Coventry University, Coventry, UK; 3 Institute of Applied Health Research, University of Birmingham, Room 124, Birmingham, B15 2TT UK

**Keywords:** Pregnancy, Maternal health, Childbirth, Mindfulness, Labour, Perinatal

## Abstract

This systematic review aims to assess the effect of mindfulness-based interventions carried out during pregnancy exploring mindfulness and mental health outcomes. A systematic review was conducted to appraise the current literature on the subject area. Inclusion and exclusion criteria were agreed and after reviewing titles, abstracts and full papers, 14 articles met the inclusion criteria and were included in the review. The quality of included articles was checked using the Quality Assessment Tool for Quantitative Studies. Pooled results of the randomised controlled trials (RCTs) reporting outcomes on anxiety, depression and perceived stress indicated no differences between the mindfulness intervention group and the control group. Pooled results of the non-RCTs reporting anxiety, depression and perceived stress showed a significant benefit for the mindfulness group. Mindfulness as an outcome was assessed in four RCTs for which the pooled results show a significant difference in favour of the mindfulness intervention when compared to a control group. The pooled results of the four non-RCTs also indicate a significant difference following mindfulness intervention. Results suggest that mindfulness-based interventions can be beneficial for outcomes such as anxiety, depression, perceived stress and levels of mindfulness during the perinatal period. Further research would be useful to explore if such benefits are sustained during the post-natal period.

**Electronic supplementary material:**

The online version of this article (doi:10.1007/s12671-017-0726-x) contains supplementary material, which is available to authorized users.

## Introduction

Pregnancy and childbirth are some of the most significant, exciting and scary experiences that a woman will experience in her lifetime. The experiences and mental health of the woman during pregnancy and throughout the post-pregnancy period are of utmost importance for the well-being of both the mother and her child. Depression or anxiety in pregnancy has been associated with an increase in obstetric complications including stillbirth, low birth weight infants, postnatal specialist care for the infant and susceptibility to more adverse neurodevelopmental outcomes including behavioural, emotional and cognitive problems (Bonari et al. 2004; Glover 2011; Talge et al. 2007). Anxiety and stress during pregnancy have been linked with premature delivery, low birth weight, and neonatal morbidity and mortality (Dole 2003; Maina et al. 2008).

While some women welcome the challenges of childbirth, others may feel a significant amount of anxiety and concern (Escott et al. 2004; Huizink et al. 2004). Anxiety and stress in pregnancy have been found to be associated with gestational length, with increases in stress and anxiety leading to preterm delivery and declines in stress and anxiety resulting in delivery at term (Glynn et al. 2008; Schetter and Tanner 2012). Preterm birth has adverse implications for foetal neurodevelopment and child outcomes and is the leading cause of infant mortality and morbidity (Schetter and Tanner 2012; Wadhwa et al. 2011). The management of anxiety in pregnant women is therefore of importance to prevent poor outcomes for both the mother and child.

Evidence suggests that up to 20% of women are affected by depression during pregnancy, and during the post-partum period, which indicates a need to support women from pregnancy to post-partum (Evans et al. 2001; Liberto 2012; Rich-Edwards et al. 2006). Untreated maternal depression can lead to illness persistence and an increase in symptom severity (Robertson et al. 2003).

Pregnancy is a key time to be caring for the mothers’ mind and mental attitude (Donegan 2015). One way in which this can be supported is through mindfulness, known to promote emotional positivity and stability. Kabat-Zinn (1994) described mindfulness as “paying attention in a particular way: on purpose, in the present moment, and nonjudgmentally” (p. 4). The process of accepting things as they are and approaching situations with an open mind reduces tension and fear and increases trust. Mindfulness can offer support to a mother both during the perinatal period and beyond (Cohen 2010; Fisher et al. 2012). Mindfulness-based interventions show promise in addressing a number of adverse outcomes, such as antenatal depression and anxiety, providing pregnant women with more empowerment and satisfaction with labour (Fisher et al. 2012). While planning for birth can be a positive experience and has its advantages, this should be flexible because if a birth plan does not come together, it could cause distress and strain on the body (Sparkes 2015). It is therefore important to focus on moment-to-moment changes, allowing some trust in the body.

Mindfulness-based interventions allow the development of abilities that are important for pregnant women and new mothers (Hall et al. 2015). These interventions encourage practice of awareness and acceptance of one’s thoughts, emotions and body sensations, building stress tolerance, reducing reactivity and avoidance of uncomfortable experiences. The seven-attitudinal factors covered in mindfulness-based interventions include non-judging, patience, beginner’s mind, trust, non-striving, acceptance and letting go (Kabat-Zinn 1990).

Pregnant women may require support through their pregnancies with mindfulness-based interventions having been suggested as potentially beneficial to support these women. The aim of this systematic review is therefore to appraise the current available literature and assess the effect of mindfulness-based interventions carried out during pregnancy in mindfulness levels and mental health-related outcomes (i.e. anxiety, stress and depression).

## Method

The systematic review protocol was registered with PROSPERO: CRD42016032627.

### Search Strategy

A search was conducted to review the current literature on mindfulness-based interventions carried out during pregnancy. A search strategy was developed using a combination of both indexing and free text terms (see Supplementary Material 1 for sample search strategy). Electronic databases including the Cochrane Library (Wiley) (including CDSR, DARE, HTA and CENTRAL), MEDLINE (Ovid), MEDLINE in Process (Ovid), EMBASE (Ovid), Science Citation Index (Web of Science) and Conference Proceedings Citation Index (Web of Science) were initially searched from their inception to 11 February 2016, with no restriction on language, and updated up to 20 February 2017. Hand searching of reference lists of relevant studies and reviews was carried out. Literature search results were uploaded to and managed using Mendeley Desktop 1.16.1 software.

### Study Selection

Two reviewers (AD, RD) independently screened the titles and abstracts of all retrieved citations. The following inclusion and exclusion criteria were applied to the citations identified by the literature search. Inclusion criteria were as follows: (i) pregnant females; (ii) mindfulness-based interventions (i.e. practices that incorporated the use of mindfulness including mindfulness-based yoga, mindfulness-based cognitive therapy, mindfulness-based stress reduction, acceptance and commitment therapy); (iii) comparative study design including randomised controlled trials (RCTs) and pre-post intervention studies and (iv) report of mental health-related outcomes (i.e. anxiety, stress, depression, levels of mindfulness). Exclusion criteria were as follows: (i) reviews or guidance papers that do not present original work; (ii) case reports; (iii) not including pregnant females or (iv) not mindfulness-based interventions. Where inclusion or exclusion could not be determined from the abstracts, full papers were retrieved. Full papers for studies deemed potentially relevant by the screening were retrieved. Disagreements were resolved through discussion and consensus between the reviewers; if consensus was not reached, a third reviewer (ES) was consulted.

### Quality Assessment

The quality of included articles was checked using the Quality Assessment Tool for Quantitative Studies (Effective Public Health Practice Project 1998). This tool, recommended by Cochrane, covers any quantitative study design and assists reviewers to score study quality. The tool evaluates aspects such as confounding, where reviewers indicate whether confounders were controlled for in the design (i.e. if participants receive an unintended intervention that may have influenced the outcomes) and intervention integrity (i.e. whether the method of measuring the intervention is the same for all participants). The tool has been judged to be suitable to use in systematic reviews of effectiveness (Deeks et al. 2003). Two reviewers conducted the assessment independently. Disagreement were resolved by discussion and consensus and if necessary consultation of a third reviewer.

### Data Extraction

For each included study, the data was extracted by one reviewer (AD) and checked for accuracy by a second reviewer (RD). Disagreements were resolved by discussion and if necessary consultation of a third reviewer. The data extracted included (1) general information including study ID, author, year, journal, study design and setting; (2) recruitment details, sample size, demographic characteristics (age, gender) and baseline health data; (3) nature of intervention, treatment duration, follow-up and (4) outcome measures. Where necessary and if possible, study authors were contacted for missing data by email. In case of no response, the authors were contacted at least twice over a 2-week period by email.

### Data Analyses

A narrative synthesis was included as the review obtained information from a diverse body of evidence. Statistical pooling of the results was carried out using RevMan 5.2. For continuous variables, the use of the mean difference (MD) or standardised mean difference (SMD) in the analysis was determined by whether studies all report the outcome using the same scale (MD) or using different scales (SMD). A random effects model was used for the meta-analyses. For non-randomised controlled trials, meta-analyses of adjusted estimates were performed as an inverse-variance weighted average.

## Results

**Fig. 1:**
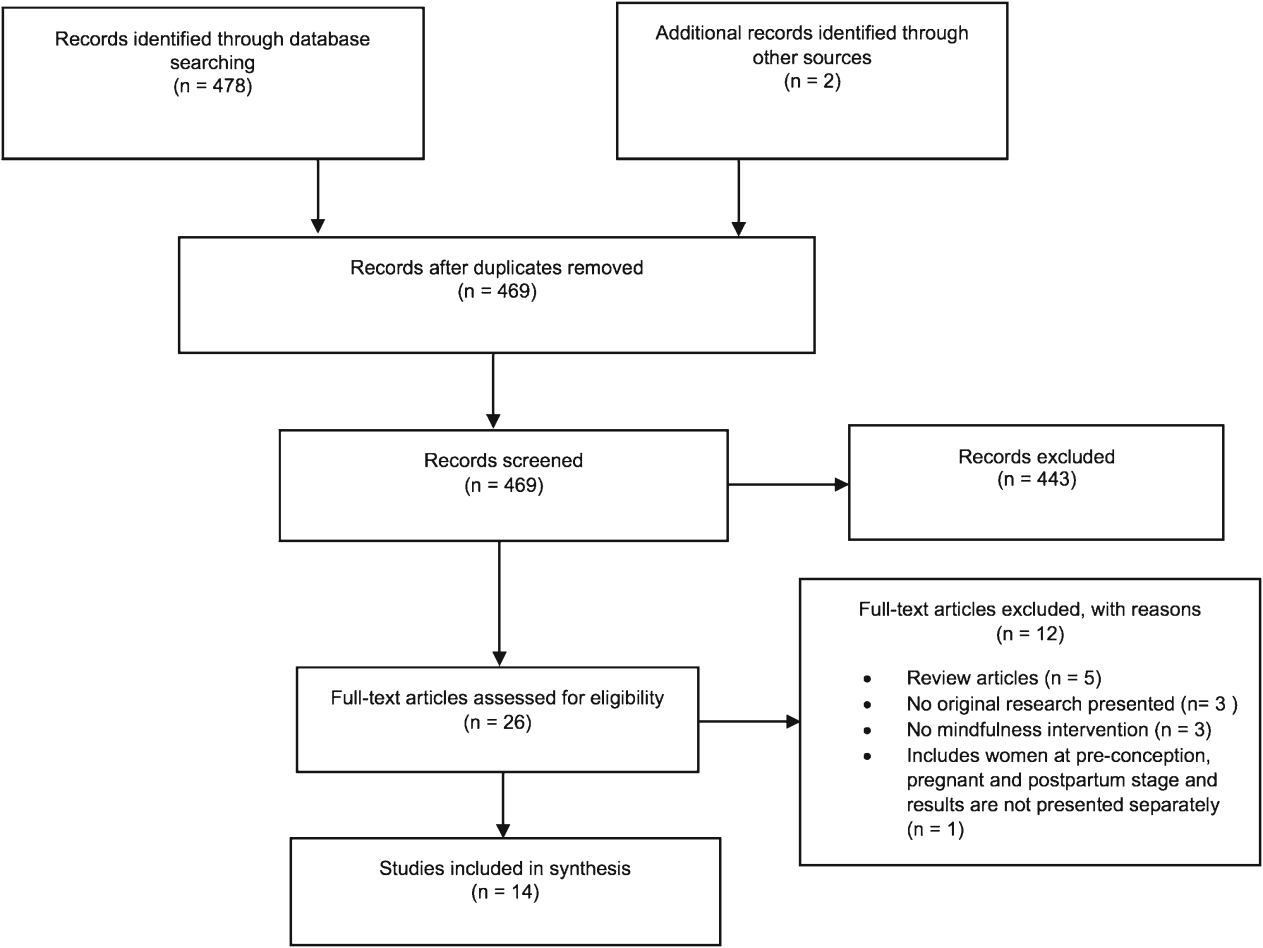
PRISMA flowchart detailing the study selection process

The search resulted in 469 abstracts screened and 26 papers identified as potentially relevant (Fig. 1). After reading the full-text, 12 articles were excluded for the following reasons: review articles (Arendt and Tessmer-Tuck 2013; Field 2008; Khianman et al. 2012; Marc et al. 2011; Smith et al. 2011), no original research presented (Beattie et al. 2014; Beddoe and Lee 2008; Donegan 2015), no mindfulness intervention (Curtis et al. 2012; Kim et al. 2008; Koyyalamudi et al. 2016), includes women at pre-conception, pregnant and postpartum stage and results are not presented separately (Miklowitz et al. 2015). Fourteen articles were found to meet the inclusion criteria. One of the articles included (Woolhouse et al. 2014) presented two studies, a one-group cohort study and a pilot RCT.

Characteristics of included studies are listed in Table 1. Prenatal or obstetric clinics and hospitals were the main source of participant recruitment. The duration of the mindfulness-based interventions varied from 6 to 9 weeks (Table 2). All of the studies carried out assessments at baseline and post-intervention. Some studies also collected post-partum data; however, the timings of post-partum assessment were variable between the studies ranging from 4 weeks to 6 months, therefore limiting the interpretation of the results. For that reason, post-partum data was not included in the meta-analyses. A number of patient reported outcome measures were used across the studies with the following measures being more commonly used in this population: Mindfulness Attention Awareness Scale (MAAS), State-Trait anxiety Inventory (STAI), Depression, Anxiety Stress Scales (DASS-21), Edinburgh postpartum Depression Scale (EPDS) and the Perceived Stress Scale (PSS). The overall global rating for quality using the using the Quality Assessment Tool for Quantitative Studies detailed in Supplementary Material 2 was moderate (*n* = 1) or weak (*n* = 14).

**Table 1:** Summary of findings from studies assessing the effect of a mindfulness-based intervention in preparation for birth

Study	Design	Setting	Population	Intervention(s)	Outcome measures	Results	Quality rating
Beddoe et al. (2009)	Cohort study (pilot)	USA—participants recruited from prenatal care providers and county-sponsored perinatal programmes.	16 healthy pregnant nulliparous women with singleton pregnancies between 12 and 32 weeks gestation at time of enrolment Mean age of 30.4 years	7-week mindfulness-based yoga group. Combined elements of the yoga methods of Iyengar (1979) and the curriculum of mindfulness-based stress reduction (MBSR). 1 session per week, each session lasting 75 min.	Acceptability Modified version of Brief Pain Inventory (BPI) Perceived Stress Scale (PSS) Physiological stress—cortisol levels in saliva (3 consecutive mornings at baseline and post-intervention) Prenatal Psychosocial Profile stressor subscale (PPP) State Anxiety -Short-form STAI (STAI-S) State-Trait Anxiety Inventory (STAI-T)	Significant decrease in stress (*p* = 0.05). Trait anxiety decreased significantly post intervention (*p* = 0.03). Time-by-group effect—overall BPI scale (*p* = 0.04), pain interference subscale (*p* = 0.04). 2nd-trimester women had significantly lower BPI scores (*p* = 0.02) after the intervention and less pain interference after intervention (*p* = 0.05) compared with 3rd-trimester group. Pain intensity remained higher after the intervention for 3rd-trimester women compared with 2nd-trimester women (*p* = 0.01). After the intervention, the 3rd-trimester group still reported significantly more hours of pain than 2nd-trimester women (*p* = 0.05). Average morning salivary cortisol level increased from baseline (*p* < 0.01).	Weak
Bowen et al. (2014)	Cohort study	Canada—participants recruited from doctors’ offices.	38 women in 15–28 weeks of gestation recruited to antenatal psychotherapy groups—interpersonal (*n* = 18) or mindfulness-based therapy (*n* = 20)	6-week group—interpersonal therapy (IPT) or mindfulness-based therapy (MFB)	Anxiety—State Trait Anxiety Inventory (STAI) Cambridge Worry Scale (CWS) Edinburgh postpartum Depression Scale (EPDS) Maternity Social Support Scale (MSSS)	There was a significant decrease in anxiety symptoms in IPT and MFB groups from intake to postpartum *p* = 0.002. Significant decrease in depression symptoms in IPT and MFB (*p* < 0.001). Worry also decreased (*p* < 0.001) from intake to the end session of the groups and into the postpartum period.	Weak
Byrne et al. (2014)	1-group cohort study (pilot)	Australia—participants recruited from birth centres, organisations that offered prenatal education, newspaper articles, online pregnancy forums and email lists.	12 pregnant women (18–28 weeks gestation) and their support companions Mean age of 30.1 years	8 weeks mindfulness-based childbirth education One 2.5-h session per week	Depression, Anxiety Stress Scales (DASS-21) Edinburgh Post Natal Depression Scale (EPDS) Efficacy Inventory Fear of childbirth before labour—Wijma delivery expectancy questionnaire Mindfulness Attention Awareness Scale (MAAS) Self-efficacy and birth outcome expectancies—childbirth and self	Significantly more self-efficacy, more positive expectations of their births and less fearful of giving birth after completing the programme. Self-efficacy and fear of birth were significant (*p* = 0.006.). Mindfulness and depression showed improvement after the programme, but results were not statistically significant. No improvements observed for depression.	Weak
Dimidjian et al. (2015)	1-group cohort study	USA—participants recruited from obstetric care.	49 pregnant women with depression histories (up to 32 weeks gestation) Mean age of 31.83 years	8 sessions—mindfulness-based cognitive therapy for perinatal women (MBCT-PD) One 2-h session per week	Client Satisfaction Questionnaire Edinburgh Postpartum Depression Scale Longitudinal Interval Follow-up Evaluation (LIFE) MBCT Adherence Scale	Significant decrease in depressive symptom levels (*p* = 0.0037) sustained throughout the perinatal period, with on-average reduction in EPDS scores relative to baseline of 2.02 (SE = 0.813) during pregnancy and postpartum (*p* = 0.013).	Weak
Dimidjian et al. (2016)	RCT (pilot)	USA—participants recruited through liaison with obstetric clinics.	86 pregnant women up to 32 weeks gestation meeting the criteria for prior major depressive disorder. Mean age of 30.98 years in the intervention group and 28.72 years in the control group.	8 sessions—mindfulness-based cognitive therapy for perinatal depression (MBCT-PD) Sessions were approximately 2 h in length and held weekly. Participants randomised to either MBCT-PD (*n* = 43) or to usual care (*n* = 43).	Acceptability Depression—Longitudinal Interval Follow-up Evaluation (LIFE) Depression severity—Edinburgh Postpartum Depression Scale (EPDS) Instructor adherence—MBCT Adherence Scale Participant satisfaction—Client Satisfaction Questionnaire Service utilisation	Level of satisfaction associated with MBCT-PD was significantly higher than that reported by participants assigned to usual care (*p* < 0.0001). There was a significant difference between groups at the post-intervention assessment for depression severity (*p* = 0.002) with participants assigned to MBCT-PD reporting significant reduction in average severity relative to baseline (*p* = 0.043), and those assigned to TAU reporting significant increase in average severity relative to baseline (*p* = 0.014). There were no differences between the groups for service utilisation (pharmacy dispensing data and psychotherapy data). The mean score on the modified MBCT Adherence Scale was 1.45 (SD = 0.17), indicating above adequate instructor adherence to the protocol.	Moderate
Duncan and Bardacke (2010)	1-group cohort study (pilot)	USA—urban context.	27 pregnant women participating in MBCP during their 3rd trimester of pregnancy (12–28 weeks gestation) Mean age of 34.61 years	9 weeks Mindfulness-Based Childbirth and Parenting (MBCP) programme 3-h sessions, once a week	Depression Scale (CES-D) Differential EAMOTIONS Scale (DES) Expanded version of Ways of coping (WOC) Five Factor Mindfulness Questionnaire (FFMQ) Perceived stress scale Pregnancy anxiety scale The Positive and Negative Affect schedule (PANAS)	Means for perceived stress, pregnancy anxiety, depression, mindfulness and the frequency and intensity of positive and negative affect were either statistically significant (*p* = 0.05) or represented a marginal trend towards significance (*p* values between 0.051 and 0.062).	Weak
Dunn et al. (2012)	Cohort study (pilot)	Australia—participants recruited from Women’s and Children’s Hospital.	10 females between 12 and 28 weeks gestation Mean age of 35.33 years Control group (*n* = 9) between 17 to 29 weeks of gestation, mean age 27.67 years	8-week mindfulness-based cognitive therapy group	Depression, anxiety and stress scale (DASS-21) Edinburgh postnatal depression scale (EPDS) Mindful attention and awareness scale (MAAS) Self compassion scale (SCS)	3 of 4 treatment group participants (75%) experienced a clinically reliable decrease in stress symptoms from baseline to post-treatment, with at least 1 participant reporting a reliable change on the majority of measures. In contrast, there was very little change in outcome scores within the control group. Post-partum outcomes indicate that as many as 67% of the treatment group participants experienced a positive change in their levels of stress and self-compassion, and half the participants reported a positive change in their depression scores as measured by the EPDS.	Weak
Goodman et al. (2014)	1-group cohort study (pilot)	USA—participants recruited from prenatal clinic of a large urban teaching hospital, local obstetric and mental health providers.	24 women 1 to 27 weeks gestation Mean age of 33.5 years	8-week CALM Mindfulness-Based Cognitive Therapy One 2-h session per week	Anxiety severity—GAD-7 Clinical anxiety—Beck Anxiety Inventory (BAI) Depression Patient Health questionnaire (PHQ-9) Depressive symptoms—Beck Depression Inventory (BDI-II) Mindfulness—Mindfulness Attention Awareness Scale Psychiatric diagnoses—Mini International Neuropsychiatric Interview (MINI) Self compassion—Self compassion Scale (SCS) Worry—Penn state worry questionnaire (PSWQ)	Statistically significant improvements (*p* < 0.01) on the BAI, PSWQ, BDI-II and SCS	Weak
Guardino et al. (2014)	RCT (pilot)	USA—participants recruited from university clinic.	47 women enrolled between 10 and 25 weeks gestation Mean age of 33.13 years	6 weekly mindfulness-based intervention Participants randomised to either a series of weekly Mindful Awareness Practices classes (*n* = 24) with home practice or to a reading control condition (*n* = 23).	Anxiety—State-Trait anxiety Inventory (STAI) Mindfulness—Five Factor Mindfulness Questionnaire Perceived stress—Perceived stress scale Pregnancy related anxiety (PRA) Pregnancy specific anxiety (PSA)	Significantly larger decrease in PSA scores in the mindfulness group (*p* < 0.05) than in the control group (*p* < 0.05). Significant decrease in PSA from baseline to 6-week follow-up in both the intervention group (*p* < 0.05) and in the control group (*p* < 0.05), with no significant difference between the 2 groups. Marginally significant group × time interaction in the model predicting change in PRA scores over time, *p* = 0.07. Post hoc analyses showed a significant decrease in PRA scores in the mindfulness group (*p* < 0.05) but not in the control group) between time 0 and time 1. However, these effects were not sustained through time 2 assessment; PRA significantly decreased in both the intervention (*p* < 0.05) and control groups (*p* < 0.05) from baseline to 6-week follow-up. No significant group × time interactions were found in models predicting change in PSS, STAI or FFMQ (all *p* < 0.10). Significant main effect of time in the FFMQ, *p* < 0.0001, *p* < .05; STAI *p* = 0.001; and *p* = .001 models, such that both groups experienced significant decreases in perceived stress and general anxiety and significant increases in mindfulness from time 0 to time 1 and from time 0 to time 2.	Weak
Matvienko-Sikar and Dockray (2016)	RCT (pilot)	Ireland—recruitment took place in the antenatal outpatient department and a private consultant clinic. Posters and leaflets were made available in various locations including general practitioner surgeries, medical centres and birth classes.	46 women between 10 and 22 weeks pregnant at recruitment Mean age of 33.87 years	Online intervention involving a gratitude diary component and a mindfulness listening component 4 times a week for 3 consecutive weeks. Participants randomised to a body scan and reflection intervention (*n* = 32) or to usual care (*n* = 14).	Depression—Edinburgh Postnatal Depression Scale (EPDS) Distress—Prenatal Distress Scale (PDQ) Grateful disposition—Gratitude during Pregnancy Scale (GDP) Mindfulness Attention Awareness Scale (MAAS) Satisfaction with life—Satisfaction with Life Scale (SWLS)	Significant decrease in stress (*p* = 0.04) between groups was observed in the mindfulness group when compared to the control group. There were no significant intervention effects for any secondary outcome measures. A significant effect of time was observed for mindfulness (*p* = 0.04), depression (*p* = 0.03) and satisfaction with life (*p* = 0.001) suggesting that depression reduced over time, and mindfulness and satisfaction with life increased over time, irrespective of experimental condition. No changes were observed for gratitude levels.	Weak
Muthukrishnan et al. (2016)	RCT	India—participants recruited from Hospital Department of Obstetrics and Gynaecology.	74 pregnant women of 12 weeks gestation Mean age of 23 years in the control group and 21 years in the 34 women in the intervention group	Mindfulness meditation programme administered 2 sessions per week for 5 weeks Participants randomised to either a mindfulness programme (*n* = 37) or usual obstetric care (*n* = 37).	Autonomic function tests (i.e. heart rate response to immediate standing, standing to lying ratio, heart rate variability and cold pressor test. Perceived stress—Perceived stress scale	There were significant decreases in perceived stress score (*p* < 0.001), blood pressure response to cold pressor test (*p* < 0.001) and systolic blood pressure response to mental arithmetic (*p* < 0.001) and a significant increase in heart rate variability (*p* < 0.001) in the study group compared to the control group.	Weak
Shahtaheri et al. (2016)	RCT	Iran—participants who were referred to a hospital maternity ward.	30 pregnant women diagnosed with depression and stress	Mindfulness-based stress reduction programme and conscious yoga in 8 weekly group session	Depression—Hamilton depression scale Quality of life—Short Form 36 Perceived stress—Perceived stress scale	Significant differences were observed between the groups for the variable general health in the quality of life tool (*p* = 0.036). Significant differences between the groups were observed for depression (*p* = 0.0001) and perceived stress (*p* = 0.0001) in favour of the mindfulness intervention.	Weak
Vieten and Astin (2008)	RCT (pilot)	USA—patients recruited from physicians’ offices, childbirth education classes.	31 women in the 2nd and 3rd trimesters who were between 12 and 30 weeks gestation at the start of the intervention Mean age of 33.9 years	8 week mindfulness-based cognitive therapy 2 h, weekly	Affect regulation Anxiety—State-trait Anxiety Inventory (STAI) Depression—(CES-D) Mindfulness Attention Awareness Scale (MAAS) Perceived stress—Perceived Stress scale (PSS) Positive and negative affect—Positive and negative affect schedule extended (PANAS-X)	At the post intervention (3rd trimester) assessment, women participating in the mindfulness group showed statistically significant decreases in state anxiety (*p* < 0.05) and negative affect (*p* < 0.04) compared with wait-list. Women participating in the mindfulness group that showed statistically significant changes in the expected direction were observed in the intervention group on all other variables. While changes in mindfulness increased somewhat from 5 to 9% at 3-month follow-up, between-group changes remained non-significant (*p* = 0.07).	Weak
Woolhouse et al. (2014)	1-group cohort study and RCT (pilot)	Australia—participants recruited from hospital antenatal clinic, childbirth education and physiotherapy classes.	20 women were recruited to the non-randomised trial, and 32 to the RCT Mean age of 32.89 years	1-group cohort 8 week mindfulness-based intervention (*n* = 13); wait-list control group (*n* = 18) RCT (pilot) Mind-Baby-Body programme is a 6-session mindfulness-based group therapy programme 2 h weekly, 6 weeks	Centre for epidemiologic studies depression scale revised (CES-D) Depression Anxiety and Stress Scale-21 (DASS) Five factor mindfulness questionnaire (FFMQ) State-Trait Anxiety Inventory, (STAI) Perceived Stress Scale (PSS)	1-group cohort Significant improvements were noted on the DASS-21 depression scale *p* = 0.01; CES-D *p* = 0.04 and the STAI state scale *p* = 0.04. Stress scores were reduced at post-programme, but the difference was not statistically significant. Mindfulness scores increased significantly on 2 of the 5 FFMQ subscales: acting with awareness *p* = 0.01 and describing *p* = 0.02. RCT (pilot) For the intervention group, all post programme mental health scores improved, with changes on the DASS-21 anxiety subscale reaching statistical significance *p* = 0.02; On the FFMQ, the intervention group showed significant increases on 2 of the 5 subscales of the FFMQ: observing *p* = <0.001 and describing *p* = 0.03. No significant changes on outcome measures over time were observed in the control group. A between-group comparison of the post-programme means for the intervention and care as usual group was conducted via 2-sample *t* tests, and no significant between-group differences were found.	Weak

**Table 2:** Characteristics of the mindfulness-based interventions used in the included studies

Study	Mindfulness-based intervention
Beddoe et al. (2009)	7 weeks, mindfulness-based yoga intervention combined elements of the yoga methods of Iyengar and the curriculum of mindfulness-based stress reduction (MBSR), a relaxation and stress management programme developed by Kabat-Zinn. The primary author, who has studied Iyengar yoga for 20 years, received extensive training in MBSR and has taught MBSR since 2002, facilitated the intervention. An aim of the intervention was to maintain fidelity with MBSR’s emphasis on mindfulness. The intervention in this study differed from MBSR in its focus on principles of Iyengar yoga, a form of postural yoga that emphasises the use of props to attain particular poses, careful anatomic alignment and correct muscular actions. In weekly sessions, mindfulness meditation skills were taught to help participants discover relationships between mindful practice and ability to cope more effectively with stress using the following techniques: (a) body scan, a progressive relaxation in which participants direct attention and observe sensations; (b) sitting meditation, involving observation of one’s breathing, sensations, emotions, sound and thoughts; (c) postural yoga, involving gentle physical poses integrated with breathing to develop strength, flexibility and balance, no more strenuous than a 30-min walk on flat ground and (d) walking meditation, involving slow and observant walking. The sessions also explored use of mindfulness in daily life, the psychological and physiological effects of stress and the possibilities of using mindfulness during birth.
Bowen et al. (2014)	The mindfulness-based groups included instruction in mindfulness, with an adaptation to increase body awareness and a greater sense of peace and acceptance of the changing body, greater awareness of emotional patterns and mental states specifically related to their pregnancy, and strategies to find more understanding and compassion for themselves. The group was process-oriented with time to connect with other women and discuss the particular challenges they were facing at that time related to their pregnancy or other issues.
Byrne et al. (2014)	The Mindfulness-Based Childbirth Education (MBCE) protocol was developed specifically for this study and included participants as active learners through group work, role play and decision-making practice using the BRAIN (benefits, risks, alternatives, intuition, nothing) model for decision-making, as well as incorporating daily mindfulness meditation homework. The programme ran over 8 consecutive weeks; each session was approximately 2.5 h. Pregnant women and their birth support partners (e.g. husband, partner, mother, friend) attended. Participants had homework CDs with mindfulness meditation instructions and a workbook to use during the week between sessions. Daily practice of techniques learned was encouraged. In addition, participants had assigned reading, usually related to prenatal information content. Each session was run by the principal investigator, a qualified childbirth educator, specialist antenatal yoga teacher and mindfulness meditation teacher. Sessions were co-facilitated by an assistant who was a registered yoga and meditation teacher. Prenatal education and mindfulness were incorporated into each session. The education part of the programme provided women and their support persons with the knowledge and skills to assist in making informed choices regarding their pregnancy, birth and parenting. To meet this aim, participants were provided with evidence-based information regarding their choices. Participants engaged in a wide range of learning activities (group discussion, role play, problem-solving activities) to prepare for birth and early parenting and were taught mindfulness exercises/meditations to develop nonreactive, present-moment awareness. They learned how to apply the practice of mindfulness to discomfort during pregnancy, labour pain and early parenting.
Dimidjian et al. (2015)	8 session groups were delivered consistent with the standard MBCT treatment manual, with modifications for the perinatal period. The standard MBCT programme includes psychoeducation and training in cognitive behavioural and mindfulness meditation practices designed to prevent depressive relapse/recurrence and promote wellness. Specifically, participants learn formal mindfulness practices (i.e. sitting and walking meditation, body scan and yoga stretching), informal mindfulness practices (i.e. mindfulness of daily activities and the 3-min breathing space) and cognitive behavioural skills (i.e. monitoring pleasant and unpleasant events, identifying thoughts and beliefs and their relationship to emotion, identifying relapse signs and developing action plans). Modifications for perinatal depression focused primarily on increased attention to brief informal mindfulness practices (e.g. washing dishes and driving), mindfulness and yoga practices customised for the perinatal period (e.g. “being with baby” informal practice and prenatal yoga poses), psychoeducation about perinatal depression and transition to parenthood and self-compassion, self-care and social support. Audio-recorded files were provided each week to guide mindfulness meditation practices at home (recorded for the study by an expert meditation teacher) and a DVD was provided to guide yoga practice (recorded for the study by an expert perinatal yoga teacher).
Dimidjian et al. (2016)	Mindfulness-based cognitive therapy for perinatal depression. The 8-session protocol for MBCT-PD was based on the standard MBCT treatment manual and theory that proposes that individuals with histories of depression are vulnerable during dysphoric states, during which maladaptive patterns present during previous episodes are reactivated and can trigger the onset of a new episode. The standard MBCT protocol was modified for use in the context of pregnancy and in anticipation of the postpartum. Modifications included stronger emphasis on brief informal mindfulness practices, given our developmental work that suggested that barriers of time, energy and fatigue are significant among pregnant women, and perinatal-specific practices. Loving kindness meditation practice was included based on the authors’ developmental work suggesting that self-criticism is a common theme among at-risk pregnant and postpartum women and that connection with one’s child is a powerful motivator for learning and practice. Loving kindness meditation practice asks women to direct awareness and positive intention at both the self and the baby through the repetition of specific phrases (e.g. “May I/my baby be filled with loving kindness. May I treat myself/my baby with kindness in good times and in hard times. May I/my baby be well and live with ease.”). Psychoeducation about perinatal depression, anxiety and worry, which are often co-occurring with depression among perinatal women, and the transition to parenthood was also included. There was an emphasis on self-care practices and cognitive–behavioural strategies to enhance social support. For example, women were guided to identify specific actions to enhance well-being during the postpartum period (e.g. reduced responsibility for family meals), to observe and decenter from self-judgments that may interfere with such actions (e.g. “good mothers can handle a new baby and making dinner”) and to role play asking for support from friends or family to carry out the specified actions. At each session, participants were given audio-recorded files to guide mindfulness meditation practices at home (recorded for the study by expert meditation teacher Sharon Salzberg) and a DVD was provided to guide yoga practice (recorded for the study by expert perinatal yoga teacher De West). The 8-session series included class sizes ranging from 3 to 9 assigned participants. Participants were allowed to complete make-up sessions by phone. Sessions were approximately 2 h in length and held weekly. Following the 8 sessions, participants were given the option of attending a monthly follow-up class. 1 of the 2 study investigators (licenced clinical psychologists with PhDs trained by one of the MBCT founders) and a KP behavioural health provider co-led the sessions.
Duncan and Bardacke (2010)	The course is held for 3 h once a week for 9 weeks. In addition, there is a 7-h silent retreat day on the weekend between class 6 and class 7 and a reunion class 4 to 12 weeks after all the women have given birth. The recommended class size ranges from 8 to 12 expectant couples. Although the course is expressly designed for expectant couples to attend together, pregnant women without a partner or whose partner cannot attend are welcome and are invited to bring a support person, if so desired. In the programme, formal mindfulness meditation instruction is given and practised in each class. In addition, participants were asked to commit to practising meditation at home using guided meditation CDs for 30 min a day, 6 days a week, throughout the course. The teaching of mindfulness is fully integrated with the current knowledge of the psychobiological processes of pregnancy, labour, birth, breastfeeding, postpartum adjustment and the psychobiological needs of the infant. A wide variety of mind–body pain coping skills for childbirth and awareness skills for coping with stress in daily life are also included. Course materials are *Full Catastrophe Living* by Jon Kabat-Zinn, two guided meditation CDs and a workbook with selected readings and resource lists. The MBCP method of childbirth preparation is unique in its focus on teaching mindfulness meditation and the necessary commitment that participants must make to practise meditation outside of class.
Dunn et al. (2012)	8-session programme undertaken by treatment group participants based on a MBCT programme. Modifications were made to the mindful movement component of the programme to ensure they were appropriate for pregnant women. Due to the group not being promoted as a treatment for mental illness, some sections of the programme that focused specifically on depression were omitted from the programme. The class was facilitated by a consultant psychiatrist, along with a counsellor, both of whom are accredited facilitators of the MBCT programme. Session 1: automatic pilot: committing to learning how to become aware of each moment; session 2: dealing with barriers and introduction to the cognitive model; session 3: learning to take awareness intentionally to the breath; session 4: staying present, taking a wider perspective and relating differently to experience; session 5: fostering an attitude of acceptance to see what if anything needs to be changed; session 6: relating to negative thoughts; session 7: managing warning signs, mastery and pleasurable activities; session 8: review and planning for regular mindfulness practice.
Goodman et al. (2014)	8 weekly 2-h group sessions following a basic MBCT session structure. 3 groups of 6 to 12 women per group were conducted. Sessions were held in a large carpeted room at an academic institution, with yoga mats, meditation cushions, chairs arranged in a circle and healthy snacks provided. Sessions included didactic presentations, group exercises aimed at cognitive skill development, formal meditation practices and leader-facilitated group inquiry and discussion. Approximately 30–40 min of daily home practice of formal and informal mindfulness practices was assigned and encouraged between classes. MP3s of formal meditations were provided for home practice and relevant readings were provided. In addition to daily formal practice, for 5 of the weeks, participants were encouraged to also practice an abbreviated mindfulness practice (the Three-Minute Breathing Space) 3 times per day. During the last 3 weeks of the intervention, they were encouraged to also utilise the Three-Minute Breathing Space “whenever they noticed unpleasant thoughts or feelings”. The CALM Pregnancy intervention was delivered by a licenced independent clinical social worker with over 10 years experience in leading mindfulness groups and who had completed MBSR training as well as a 5-day MBCT training course. All sessions were audiotaped and reviewed for the purposes of treatment fidelity monitoring and ongoing supervision by the principal investigator, an experienced psychiatric/mental health advanced practice nurse with expertise in treating perinatal anxiety and depression and who also completed professional-level training in both MBSR and MBCT.
Guardino et al. (2014)	6-week series of 2-h group classes. The course series is entirely secular in nature and is designed to provide a comfortable atmosphere for all participants regardless of their spiritual beliefs and backgrounds. Each class series was led by 1 of 3 trained instructors following a curriculum outlined in a standardised instructor’s manual. Participants were trained in the practice of mindfulness meditation and its applications to daily life through participation in instructor-led group meditations, lectures about mindfulness practices and discussions, which allowed participants to share their experiences with one another and for the instructor to address participant questions. To record attendance, participants signed in upon entry to the classroom. At the beginning of the class series, each participant was given a compact disc with recordings of instructor-guided meditations to use at home and provided with homework assignments each week (daily meditations ranging from 5 to 17 min). Participants learned mindfulness meditation practices including sitting and walking meditations, and how to work with difficult thoughts and emotions. Prior to the start of the class series, intervention participants received 6 weekly diaries to use in recording time spent engaged in at-home mindfulness practice.
Matvienko-Sikar and Dockray (2016)	A dual component online intervention was used involving a gratitude diary component and a mindfulness listening component. Instructions for both components were tailored to incorporate aspects of prenatal experience. In the gratitude diary, participants listed up to 5 things they felt grateful for during the previous 24 h. Instructions for diary completion also made reference to an experience of pregnancy, “feeling your baby move”, as an example of something for which participants may feel grateful. The mindfulness listening component was a single mindfulness meditation audio file, the body scan that involved a guided focus on the breath and progressive sections of the body. It incorporated a focus on the pregnant belly and presence of the baby. The audio for the mindfulness body scan was produced by the researcher for the purpose of the study, building on existing mindfulness body scans, and lasted 6 min. Participants used the intervention 4 times a week for 3 consecutive weeks. The timing of intervention use was pre-specified for participants as the Monday, Wednesday, Friday and one weekend day of each study week.
Muthukrishnan et al. (2016)	The mindfulness meditation programme administered 2 sessions per week for 5 weeks. This was for explaining techniques, practice and feedback along with 30-min daily home practice. The process of framing the programme was based on the guidelines provided by Kabat-Zinn, and some adaptation done for the patients involved in the study. Week 1, session 1: welcoming and explanation of basics of mindfulness meditation; week 1, session 2: explanation of importance of mindfulness of breathing on stress reduction; week 2, session 1: explanation of mindfulness in sitting and lying posture. Practicing mindfulness breathing. Mp3 CD provided for home practice; week 2, session 2: explanation and practicing sitting and lying mindfulness of breathing. Mp3 CD provided for home practice; week 3, session 1: explanation and practicing sitting and lying mindfulness of breath, mindfulness of sounds around them and thoughts. Mp3 CD provided for home practice; week 3, session 2: practice of mindfulness of breath, sounds around them. Mp3 CD provided for home practice; week 4, session 1: explanation of stress during pregnancy and importance of bringing mind to “present moment”; week 4, session 2: explanation and practice of mindfulness walking. Explanation and practice of body scan of all parts. Mp3 CD provided for home practice; week 5, session 1: practice of mindfulness in sitting, lying and walking. Practice of body scan of all parts; week 5, session 2: summarising sessions, feedback and post session assessments.
Shahtaheri et al. (2016)	Mindfulness-based stress reduction programme and conscious yoga in 8 weekly group sessions. No further details provided.
Vieten and Astin (2008)	The intervention incorporated 3 approaches to cultivating mindfulness: (1) mindfulness of thoughts and feelings through breath awareness and contemplative practices, (2) mindfulness of the body through guided body awareness meditation and mindful hatha yoga and (3) presentation of psychological concepts that incorporate mindfulness such as acceptance and cultivation of an observing self. Each of these elements accounted for approximately one-third of the intervention. The intervention contained approximately equal parts education, discussion and experiential exercises, with more weight on education in the early sessions, and more on discussion and experiential exercises in the later sessions. Adaptations of typical mindfulness-based intervention components included (1) inclusion of awareness of the developing foetus and belly during the body scan meditation; (2) use of explanatory examples and exercises having to do with pregnancy and early parenting such as mindfulness regarding pain or sleep issues during pregnancy, anxiety about labour or dealing with a difficult-to-console infant; and (3) greater inclusion of walking and moving mindfulness practices and forms of mindful movement that have been tailored for pregnant women such a prenatal yoga. Participants were provided with weekly readings relevant to the material presented in class, as well as a compact disc with three 20-min guided meditations, which they were encouraged to utilise daily. The training was 2 h in duration per week for 8 weeks and was facilitated by a licenced clinical psychologist trained in mindfulness-based interventions, as well as a certified prenatal yoga instructor. Group sizes ranged from 12 to 20 women (and their infants in the postnatal wait-list control group), and groups were held in the multipurpose rooms of a large urban hospital, as well as a local synagogue.
Woolhouse et al. (2014)	A 6-session mindfulness-based group therapy programme developed specifically for pregnancy by one of the investigators. Participants are introduced to the mindfulness approach and strategies, including formal and informal mindfulness practices, mindful movement and cognitive exercises. The sessions took place on weekdays, on-site. Two alternative timings were offered (during work hours and in the evening). Participants did not receive any remuneration (such as travel or parking costs) for participation in the programme. Sessions ran for 2 h and occurred weekly for 6 weeks. Participants were encouraged to attend all sessions, but were considered to have completed the programme if they attended 4 of the 6 sessions. The group facilitator was a female mental health professional (psychiatrist/psychologist) with specific training in the facilitation of mindfulness groups. The facilitator was responsible for noting and responding to any emotional issues which arose for group participants during the course of the programme and for providing appropriate referral pathways where necessary. Each session included a formal meditation practice (15–20 min), a discussion of home mindfulness practices, the mindful movement sequence, a weekly discussion topic and a breathing space. Each week suggestions were given for home practice with repetition emphasised as a significant reinforcer of new skills. Week 1 included time to get to know each other, an introduction to mindfulness and a mindful breathing practice. Week 2 focused on mindfulness of the body, including a body scan, and the importance of the body in communicating with babies. Week 3 introduced ideas related to mindfulness of pain (physical and emotional), and how this might be relevant to labour. Week 4 focused on an ice meditation where participants were given experience practicing mindfulness of painful sensations. Week 5 focused on mindfulness of thoughts, and Week 6 was centred on self-compassion and the use of mindfulness skills in motherhood.

**Fig. 2:**
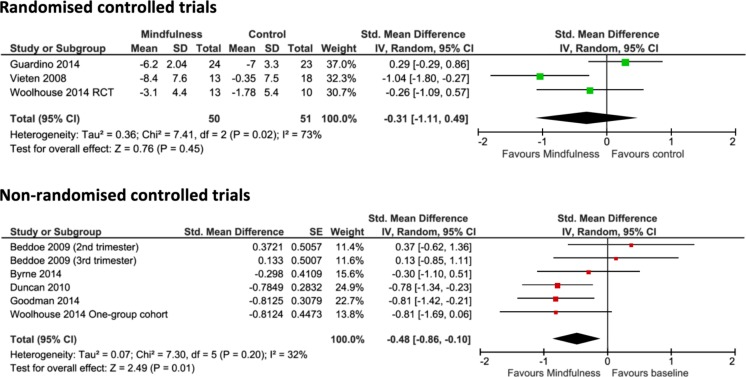
Pooled results for change in anxiety from RCTs and non-RCTs

Three RCTs reported outcomes on anxiety (Guardino et al. 2014; Vieten and Astin 2008; Woolhouse et al. 2014). The pooled results indicated no differences between the mindfulness intervention group and the control group (−0.31, 95% CI −1.11 to 0.49, *p* = 0.45) (Fig. 2). Pooled results of six non-RCTs reporting anxiety (one study investigating two trimesters with different patients) showed a significant benefit for the mindfulness group (−0.48, 95% CI −0.86 to −0.10, *p* = 0.01).

**Fig. 3:**
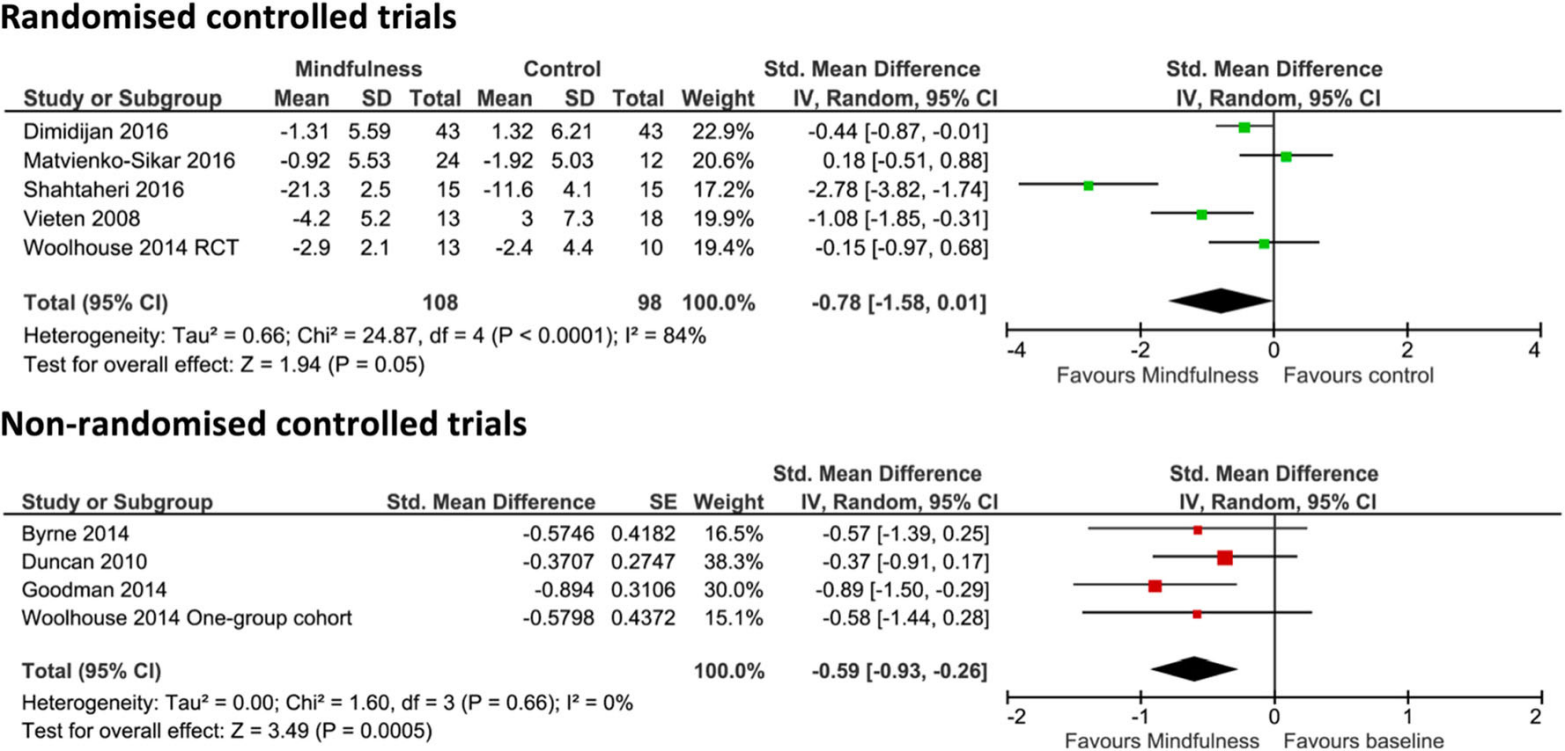
Pooled results for change in depression from RCTs and non-RCTs

The pooled results of RCTs evaluating depression indicated no difference between the mindfulness intervention group and the control group (−0.78, 95% CI −1.58 to 0.01, *p* = 0.05) (Fig. 3). Pooled results of the non-RCTs assessing depression suggested, however, a significant difference favouring mindfulness intervention (−0.59, 95% CI −0.93 to −0.26, *p* = 0.0005).

**Fig. 4:**
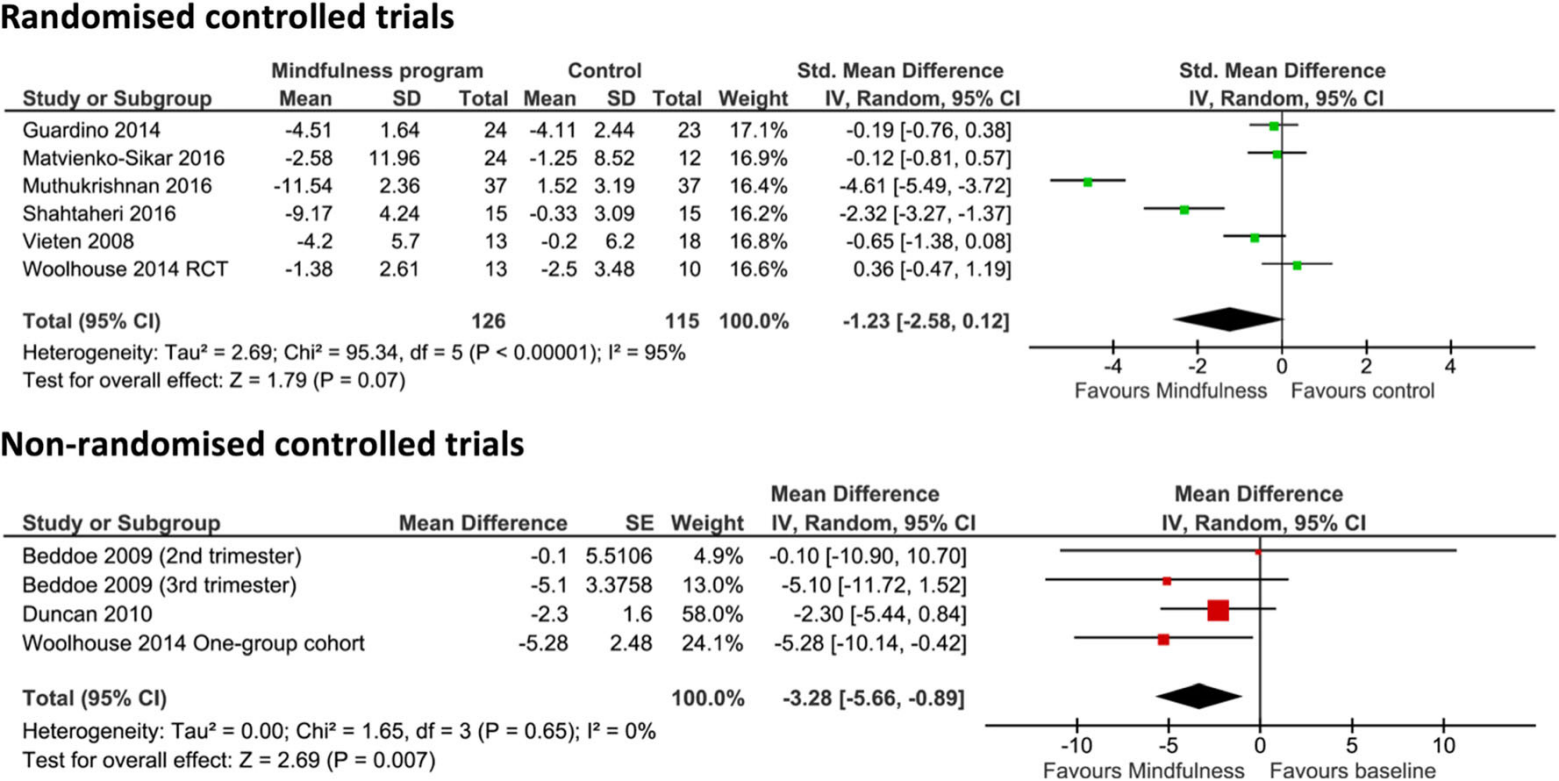
Pooled results for change in perceived stress from RCTs and non-RCTs

Perceived stress was assessed in six RCTs with pooled results demonstrating no significant differences between the mindfulness and the control group (−1.23, 95% CI −2.58 to 0.12, *p* = 0.07) (Fig. 4). The pooled results of four non-RCTs evaluating perceived stress showed a significant difference following mindfulness intervention (−3.28, 95% CI −5.66 to −0.89, *p* = 0.007).

**Fig. 5:**
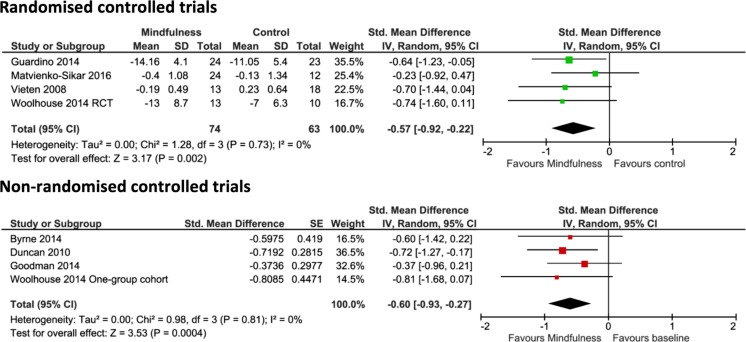
Pooled results for change in mindfulness awareness from RCTs and non-RCTs

Mindfulness as an outcome was assessed in four RCTs for which the pooled results showed a significant difference in favour of mindfulness intervention when compared to a control group (−0.57, 95% CI −0.92 to −0.22, *p* = 0.002) (Fig. 5). Consistently with this observed effect, the pooled results of the four non-RCTs also indicated a significant difference following mindfulness intervention (−0.60, 95% CI −0.93 to −0.27, *p* = 0.0004).

## Discussion

The pooled results of RCTs included in this systematic review, although suggesting a trend in favour of mindfulness interventions, did not produce statistically significant results when assessing changes in anxiety, depression and perceived stress. This may be due to the limited number of RCTs and the small numbers of participants included in these RCTs. Furthermore, the RCTs available were either pilot or feasibility studies, or not adequately powered to demonstrate the effectiveness of a mindfulness-based intervention. The results of the pooled analysis of non-RCTs, although indicating statistically significant benefits, should be interpreted with caution as the analysis was based on studies with higher risk of bias, poor design and prone to confounding. Consistent results in the pooled analysis between RCTs and non-RCTs were observed for mindfulness, therefore demonstrating an increase in awareness following a mindfulness-based intervention. The ensuing paragraphs discuss in further detail the results observed in this review in terms of the effect of mindfulness-based interventions carried out during pregnancy in the mental health-related outcomes assessed (i.e. anxiety, stress and depression) and mindfulness levels.

Previous evidence has found support for mindfulness when used to reduce anxiety and depression. A meta-analysis demonstrated that mindfulness-based therapy is a promising intervention for treating anxiety and mood problems in clinical populations (Hofmann et al. 2010). Additionally, a systematic review and meta-analysis of RCTs evaluating the effect of mindfulness-based therapy on symptoms of anxiety and depression in adult cancer patients and survivors found that mindfulness-based therapy was associated with significantly reduced symptoms of anxiety and depression from pre- to post-treatment (Piet et al. 2012). These studies demonstrate the beneficial use of mindfulness-based interventions to reduce anxiety and depression in clinical populations. As highlighted above, our pooled analysis of the RCT data suggested some benefit of using mindfulness-based interventions to reduce anxiety and depression in pregnant women. Although the results were not statistically significant and were based on either pilot, feasibility or underpowered RCTs, they do suggest that there is a need for more robust, controlled research in this area.

To further our understanding of perceived stress and the use of mindfulness in pregnancy, suggestions from other research should also be explored. According to Beddoe et al. (2009), a factor to be considered is that the differences in perceived stress between women in the second and third trimester could be due to normal changes resulting from pregnancy. Roth and Robbins (2004) have suggested that mindfulness does not only have a positive effect on stress reduction in specific populations, such as maternal health and pain, but can be beneficial for healthy individuals, emphasising mindfulness as a promising intervention to improve stress levels. Furthermore, mindfulness-based stress reduction was found to reduce ruminative thinking and trait anxiety, as well as to increase empathy and self-compassion, suggesting that mindfulness-based interventions are also able to reduce stress levels in healthy people (Chiesa and Serretti 2009). These studies support the notion that mindfulness may improve perceived stress not only in clinical populations but also in healthy individuals. However, Guardino et al. (2014) suggested that mindfulness training alone may not be sufficient to consistently reduce levels of perceived stress during pregnancy. It may therefore be of relevance to explore if mindfulness in combination with additional support/interventions may contribute to a more consistent reduction in perceived stress during pregnancy.

Levels of mindfulness were found to increase in pregnant women participating in a mindfulness intervention group and also to increase as pregnant women progressed through the intervention. Mindfulness practice has been hypothesised to develop the capacity to observe the changing mental and physiological states and sensations without necessarily trying to change them (Beattie et al. 2014). In a population with pregnant women, the individual will be more likely to accept what is happening and make clearer decisions about their response. Mindfulness benefits may include improvement in health outcomes for women and their families (Beattie et al. 2014).

This review has a number of limitations which must be considered when interpreting the results. There was considerable heterogeneity in the pooled analyses of the RCTs. The authors of two studies were contacted for additional data to include in the meta-analysis; however, no response was obtained. The results from the control groups of the RCTs may have been confounded; in the Guardino et al. (2014) study, 30% of the control group took a prenatal yoga class during their pregnancies, which could have resulted in a reduction on anxiety levels of the control group. Furthermore, the reading materials given to the control group may have also decreased their anxiety indirectly, by individuals gaining more knowledge about their pregnancy. This could suggest that people may improve on their own and not as a result of an intervention. It has been observed that participants in control conditions are often motivated to make their own improvements (McBride 2015). Additionally, Kinser and Robins (2013) found that control group participants ended up making more significant changes than was expected, due to discussions between the groups, social support and participating in other activities which promoted positive behaviour changes. Therefore, it would be useful for future research to explore mediational analyses to further understand this area.

In conclusion, the present systematic review suggests that mindfulness-based interventions may be beneficial for outcomes such as anxiety, depression, perceived stress and levels of mindfulness. However, there is a lack of evidence in this area. Further research including adequately powered RCTs is warranted to confirm the effectiveness of mindfulness in pregnant women. It would be of value to explore if benefits reported by pregnant women following a mindfulness-based intervention are of benefit during the post-natal period and to their offspring. If future evidence demonstrates that mindfulness-based programmes are effective, by recommending these interventions, health care professionals can help pregnant women to manage a number of pregnancy-related factors associated with the expectations and uncertainties of becoming a mother.

## Electronic Supplementary Material

ESM 1 (PDF 182 kb)

ESM 2 (PDF 25 kb)
