# Supplementary material for: Mindfulness-Based Interventions During Pregnancy: a Systematic Review and Meta-analysis
Source: Mindfulness (N Y). 2017 Apr 17;8(6):1421–37. doi: 10.1007/s12671-017-0726-x (PMC5693962; doi:10.1007/s12671-017-0726-x)
Supplement: Supplementary file 1 — (PDF 182 kb) [file 12671_2017_726_MOESM1_ESM.pdf]

Supplementary Material 1

Database: Ovid MEDLINE(R) In-Process & Other Non-Indexed Citations and Ovid MEDLINE(R) <1946 to Present> **20FEB2017**

Search Strategy:

----

1 exp Pregnancy/ or pregnancy.mp.
2 Midwifery.mp. or exp Midwifery/
3 Maternal Health.mp. or exp Maternal Health/
4 Perinatal Care.mp. or exp Perinatal Care/
5 Parenting.mp. or exp Parenting/
6 Parturition.mp. or exp Parturition/
7 Childbirth.mp.
8 Prenatal Education.mp. or exp Prenatal Education/ or exp Prenatal Care/
9 Patient Satisfaction.mp. or exp Patient Satisfaction/
10 obstetric delivery.mp. or exp Delivery, Obstetric/
11 1 or 2 or 3 or 4 or 5 or 6 or 7 or 8 or 9 or 10
12 Labor Pain.mp. or exp Labor Pain/
13 Psychological adaptation.mp. or exp Adaptation, Psychological/
14 Psychological stress.mp. or exp Stress, Psychological/
15 perinatal distress.mp.
16 postpartum depression.mp. or exp Depression, Postpartum/
17 separation anxiety.mp. or exp Anxiety, Separation/
18 12 or 13 or 14 or 15 or 16 or 17
19 11 and 18
20 Mindfulness.mp. or exp Mindfulness/
21 Meditation.mp. or exp Meditation/
22 Yoga.mp. or exp Yoga/
23 20 or 21 or 22
24 19 and 23

****
