# Supplementary material for: Mindfulness-Based Interventions During Pregnancy: a Systematic Review and Meta-analysis
Source: Mindfulness (N Y). 2017 Apr 17;8(6):1421–37. doi: 10.1007/s12671-017-0726-x (PMC5693962; doi:10.1007/s12671-017-0726-x)
Supplement: Supplementary file 2 — (PDF 25 kb) [file 12671_2017_726_MOESM2_ESM.pdf]

Supplementary Material 2

	Selection Bias		Study Design				Confounders		Blinding		Data Collection methods		Withdrawals and Dropouts		Global Rating
	Q1	Q2	Q1	Q2	Q3	Q4	Q1	Q2	Q1	Q2	Q1	Q2	Q1	Q2	
**Beddoe et al (2009)**	3	3	5	No	n/a	n/a	2	4	3	3	1	1	1	1	
	Weak		Moderate				Weak		Moderate		Strong		Strong		Weak
**Bowen et al (2014)**	3	5	3	No	n/a	n/a	2	4	3	3	1	1	3	4	
	Weak		Moderate				Weak		Moderate		Strong		Weak		Weak
**Byrne et al (2014)**	3	5	5	No	n/a	n/a	3	4	3	3	1	1	1	1	
	Weak		Moderate				Weak		Moderate		Strong		Strong		Weak
**Dimidijan et al (2015)**	3	2	5	No	n/a	n/a	3	4	3	3	1	1	1	1	
	Weak		Moderate				Weak		Moderate		Strong		Strong		Weak
**Dimidijan et al (2016)**	3	1	1	Yes	No	n/a	1	1	3	3	1	1	1	2	
	Weak		Strong				Strong		Moderate		Strong		Moderate		Moderate
**Duncan & Bardack (2009)**	3	5	5	No	n/a	n/a	3	4	3	3	1	1	3	4	
	Weak		Moderate				Weak		Moderate		Strong		Weak		Weak
**Dunn et al (2012)**	3	5	3	No	n/a	n/a	3	4	3	3	1	1	3	4	
	Weak		Moderate				Weak		Moderate		Strong		Weak		Weak
**Goodman et al (2014)**	3	5	5	No	n/a	n/a	3	4	3	3	1	1	1	1	
	Weak		Moderate				Weak		Moderate		Strong		Strong		Weak
**Guardino et al (2014)**	3	1	1	Yes	Yes	Yes	2	4	3	3	1	1	1	1	
	Weak		Strong				Weak		Moderate		Strong		Strong		Weak
**Matvienko-Sikar et al (2016)**	3	3	1	Yes	No	n/a	2	4	3	3	1	1	1	2	
	Weak		Strong				Weak		Moderate		Strong		Moderate		Weak
**Muthukrishnan et al (2016)**	3	5	1	Yes	No	n/a	2	4	3	3	1	1	3	4	
	Weak		Strong				Weak		Moderate		Strong		Weak		Weak
**Shahtaheri et al (2016)**	3	5	1	Yes	No	n/a	3	4	3	3	1	1	3	4	
	Weak		Strong				Weak		Moderate		Strong		Weak		Weak
**Vieten & Astin (2008)**	3	2	1	Yes	No	n/a	3	4	3	3	1	1	1	1	
	Weak		Strong				Weak		Moderate		Strong		Strong		Weak
**Woolhouse et al (2014)**															
One-group cohort study	3	4	5	No	n/a	n/a	3	4	3	3	1	1	1	3	
	Weak		Moderate				Weak		Moderate		Strong		Weak		Weak
RCT (pilot)	3	4	1	Yes	No	No	3	4	3	3	1	1	1	2	
	Weak		Strong				Weak		Moderate		Strong		Moderate		Weak

Quality Assessment for Quantitative Studies (Effective Public Health Practice Project, 2007)
